# Dopamine D1 receptor-mediated NMDA receptor insertion depends on Fyn but not Src kinase pathway in prefrontal cortical neurons

**DOI:** 10.1186/1756-6606-3-20

**Published:** 2010-06-22

**Authors:** Jian-Li Hu, Gang Liu, Yan-Chun Li, Wen-Jun Gao, Yue-Qiao Huang

**Affiliations:** 1 Department of Neurobiology and Anatomy, Drexel University College of Medicine, 2900 W. Queen Lane, Philadelphia, PA 19129, USA

## Abstract

**Background:**

Interactions between dopamine and glutamate in the prefrontal cortex are essential for cognitive functions such as working memory. Modulation of N-methyl-D-aspartic acid (NMDA) receptor functions by dopamine D1 receptor is believed to play a critical role in these functions. The aim of the work reported here is to explore the signaling pathway underlying D1 receptor-mediated trafficking of NMDA receptors in cultured rat prefrontal cortical neurons.

**Results:**

Activation of D1 receptor by selective agonist SKF-81297 significantly increased the expression of NR2B subunits. This effect was completely blocked by small interfering RNA knockdown of Fyn, but not Src. Under control conditions, neither Fyn nor Src knockdown exhibited significant effect on basal NR2B expression. D1 stimulation significantly enhanced NR2B insertion into plasma membrane in cultured PFC neurons, a process obstructed by Fyn, but not Src, knockdown.

**Conclusions:**

Dopamine D1 receptor-mediated increase of NMDA receptors is thus Fyn kinase dependent. Targeting this signaling pathway may be useful in treating drug addiction and schizophrenia.

## Background

The prefrontal cortex (PFC) plays a well-established role in working memory function [1,2] and dysfunction of either dopamine D1 receptor or N-methyl-D-aspartic acid (NMDA) receptor in this brain region has long been believed to underlie the symptoms of schizophrenia and other neuropsychiatric disorders [3-6]. Moreover, dopaminergic and glutamatergic afferents, which are from midbrain, thalamus and cortical structures, converge onto the spines of pyramidal neurons in PFC [7,8], providing the cellular basis for interactions between dopamine and glutamate signaling in the same neuron [5]. In the past decade, extensive studies have focused on the interactions between dopamine and NMDA receptors [3,6,9-11]. Studies show that dopamine influences both long-term potentiation and depression in PFC [12,13]. Moreover, some studies indicate that physical coupling and functional cross-talk may occur between NMDA and D1 receptors [5,14-16]. Despite the overwhelming evidence of the role of D1 and NMDA receptors in synaptic functions, the molecular mechanisms involved in these interactions, particularly signaling pathways in D1-mediated NMDA receptor trafficking, are still elusive.

Recent evidence indicates that NMDA receptors are not static, as traditionally believed; instead they can move into and out of synapses [17-20]. Indeed, D1 receptor activation led to rapid trafficking of NMDA receptor subunits, with increased expression of NMDA receptor subunits NR1 and NR2B in the dendrites of cultured striatal and prefrontal neurons [21,22]. The regulation of NMDA receptor trafficking is a dynamic and potentially powerful mechanism for the synaptic plasticity associated with drug addiction, Alzheimer's disease and schizophrenia [23]. Unfortunately, little is known about the signaling pathways implicated in the regulation of NMDA receptor trafficking. Previous studies have emphasized the importance of Src family kinases in this process. Src and Fyn, the major members with the highest degree of primary sequence homology, both exist in the postsynaptic density (PSD) [24] and likely upregulate NMDA receptor activity in the central nervous system (CNS) [25,26]. Activation of D1 receptors is known to enhance NMDA receptor functions. Considering the importance of Src and Fyn in NMDA receptor regulation [23,27-29], we hypothesized that Src family kinases, especially Fyn and Src, mediate D1 receptor activation-induced NMDA receptor increase in PFC. We tested this hypothesis in cultured PFC neurons and found that Fyn, but not Src, is critical in D1 receptor activation-induced NMDA receptor trafficking, particularly in surface insertion.

## Results

### Cultured prefrontal neurons express both D1 and NMDA receptors

**Figure 1:**
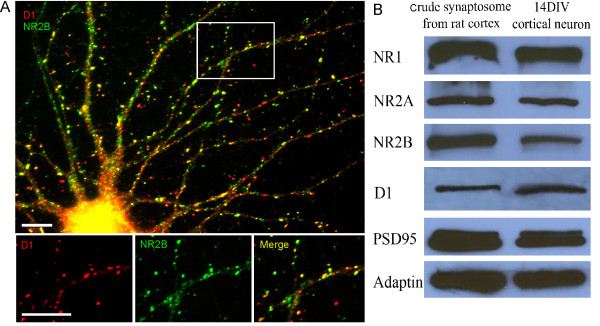
**Expression of D1 and NMDA receptors in cultured PFC neurons**. (A) Localization of D1 receptor and NR2B in cultured PFC neurons. PFC neurons in dissociated culture at 14 DIV were labeled for endogenous D1 and NR2B receptors with double immunofluorescent staining. Lower panel, images at higher magnification showing the colocalization of D1 and NR2B on the dendritic shafts and spines. Scale bars = 10 μm. (B) Analysis by Western blotting shows the protein expression of D1 receptor and NMDA receptor subunits in neuronal lysates. Crude synaptosome (20 μg of protein) from adult rat cortex (lane 1) and 14 DIV cultured PFC neurons (lane 2) were subjected to sodium dodecyl sulfate-polyacrylamide gel electrophoresis (SDS-PAGE) and probed for NR1, NR2A, NR2B, D1, PSD95, and adaptin. Both adult rat cortex crude synptosome and cultured PFC neurons exhibited similar expressions of NMDA and D1 receptors, as well as synaptic protein, suggesting the validity of the experiments in primary cultured neurons.

To characterize the expression pattern of D1 and NMDA receptors in cultured PFC neurons, we performed double immunofluorescent staining of both D1 and NR2B receptors in low-density cultures at 14 DIV. As shown in Figure 1, both D1 receptors and NR2B subunits exhibited tiny, bright puncta along the dendrites, either appearing to be associated with dendritic spines or located in the dendritic shafts (Figure 1A). The punctate stainings of D1 and NR2B subunits were largely colocalized in cultured PFC neurons. These characteristic patterns of D1 and NR2B distribution in the cultured neurons were further confirmed at the protein levels in cultured PFC neurons at 14 DIV and in adult rat PFC brain homogenate (Figure 1B). The expressions of D1 and NR2B, as well as NR1 and NR2A subunits and PSD95, appeared to be abundant and similar in prefrontal brain crude synaptosome and cultured PFC neurons (Figure 1B). These results provided solid evidence that, despite the less mature phenotype of cultured prefrontal neurons, they contain all of the components and machineries required for D1-NMDA interactions.

### D1 receptor stimulation increases NR2B expression in cultured PFC neurons

Although NMDA receptors are largely composed of NR1 (obligatory subunit), NR2A and NR2B, we focused on the effect of D1 class-mediated regulation in NR2B trafficking to test our hypothesis. This is because NR2B is the major tyrosine-phosphorylated subunit in the post-synaptic density [14,30,31] and NR2B subunits are the most dynamic and mobile subunit in the NMDA receptors [19,32]. In addition, tyrosine phosphorylation of NR2B at Tyr1472 is dependent on Src/Fyn family kinases [32]. Although NR1 is an obligatory subunit of NMDA receptors, it is known to be phosphorylated by serine kinases PKC and PKA but not by tyrosine kinases [30,33,34], whereas NR2A seems to be less affected by activation of D1 receptors [21,22].

**Figure 2:**
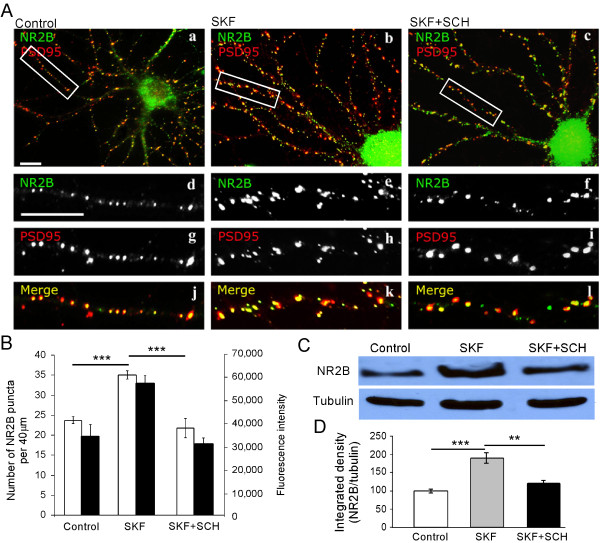
**D1 receptor agonist SKF-81297 enhances the expression and clustering of NR2B subunit**. (A) PFC neurons at 16 DIV were treated with vehicle (a, d, g, j), SKF-81297 (10 μM; b, e, h, k), or SKF in the presence of SCH (15 μM; c, f, i, l), respectively, and double labeled for endogenous NR2B (green) and PSD95 (red). Panel j, k and l are merged from respective NR2B (green) and PSD-95(red) staining. Scale bars = 10 μm. (B) Quantification of total NR2B immunofluorescence staining. White bars: NR2B puncta number; black bars: NR2B fluorescence intensity. SKF-81297 significantly increased total NR2B puncta number and immunofluorescence intensity compared with control. Results are presented as mean number and fluorescence intensity of total NR2B puncta. Statistical analysis was performed using ANOVA followed by Tukey multiple comparison test (n = 20, ** p < 0.01, *** p < 0.001). Data represent mean ± SEM. (C) Proteins were isolated at 16 DIV from cultured high-density PFC neurons treated with DMSO, D1 receptor agonist SKF-81297, or SKF-81297 + SCH-23390. The proteins were resolved on SDS-PAGE and immunoblotted for NR2B and reprobed with tubulin. (D) Quantification of NR2B protein expression in PFC neurons. SKF-81297 significantly increased NR2B expression, which was completely blocked by pretreatment with SCH-23390 (n = 4, ** p < 0.01, *** p < 0.001). Integrated intensity was measured using Image J and control protein level of NR2B was set to 100% after being normalized to tubulin. These results indicate that D1 receptor stimulation does increase NR2B expression, which may eventually increase NMDA receptor trafficking to membrane surface.

To study D1 receptor activation-mediated NR2B expression in cultured PFC neurons, the cultures were treated for 10 min with DMSO (0.1%) as vehicle control, the selective dopamine D1 receptor agonist SKF-81297 (10 μM), or the dopamine D1 receptor antagonist SCH-23390 (15 μM) followed by SKF-81297 (10 μM) for 10 min. The cultures were then restored to 37°C/5% CO_2 _in normal medium for another 15 min to allow the receptor trafficking. When the PFC neurons were treated with the D1 agonist SKF-81297 alone, expression of NR2B subunits in cultured PFC neurons was significantly enhanced compared with control, as shown in Figure 2 (puncta number: 23.8 ± 0.93 in control vs 35.0 ± 1.09 in SKF, n = 20, p < 0.001; fluorescence intensity: 33,875 ± 2439 in control vs 56,497 ± 3397 in SKF, n = 20, p < 0.01; Figure 2A and 2B). However, when cultured PFC neurons were pretreated by D1 antagonist SCH-23390 followed by SKF-81297 administration, the enhancement in NR2B expression was significantly attenuated (puncta number: 21.8 ± 1.10 in SKF + SCH, n = 25, p < 0.001; fluorescence intensity: 30,544 ± 2504 in SKF + SCH, n = 25, p < 0.001; Figure 2A and 2B). These results were further confirmed using Western blot analysis. After treatment, the homogenates from high-density cultured PFC neurons were prepared and changes in NR2B protein expression were probed by immunoblotting. SKF-81297 treatment increased NR2B protein by 91.1% ± 14.51% compared with control (n = 4; p < 0.001; Figure 2C) and the increase was specifically blocked by pretreatment of SCH-23390 (Figure 2C and 2D). In contrast, D1 receptor stimulation did not show any detectable effect on PSD95 expression (Figure 2A). Since the D1-activation induced an increase in total NR2B subunit staining, we asked whether this increase is sensitive to the protein synthesis inhibitor anisomycin (see Additional File 1). These results indicate that D1 receptor stimulation does increase NR2B expression, which may eventually increase the NMDA receptor trafficking to membrane surface.

### Fyn or Src knockdown has no effect on total NR2B expression in cultured PFC neurons

**Figure 3:**
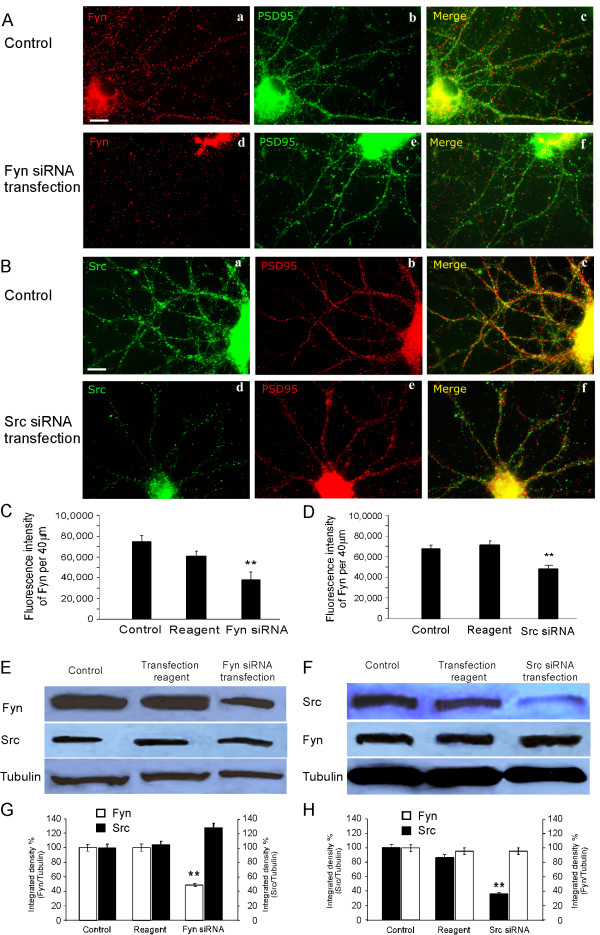
**Efficiency of Src and Fyn knockdown in cultured PFC neurons**. (A) 11 DIV PFC neurons were transfected for 48 h with Fyn siRNA and stained with anti-Fyn and PSD95. Fyn, but not PSD95, decreased dramatically after siRNA transfection. Scale bar = 10 μm. (B) 11 DIV PFC neurons were transfected for 48 h with Src siRNA and stained with anti-Src and PSD95. Similarly, Src, but not PSD95, was significantly reduced in transfected neurons. Scale bar = 10 μm. (C and D) Quantifications of Fyn and Src immunofluorescence in dendrites suggest the effective knockdown of both Fyn and Src kinases in the cultured PFC neurons (n = 30, ** p < 0.01, *** p < 0.001). (E-H) Total protein levels of Fyn and Src were determined by Western blotting after Fyn and Src siRNA transfection in cultured PFC neurons. The blots were stripped and reprobed with antitubulin as loading control and the siRNA knockdown specificities were demonstrated by re-probing the Src and Fyn, respectively. Integrated intensity analysis showed significant decreases in both Fyn and Src proteins after knockdown (n = 3, ** p < 0.01). Control protein levels of Fyn and Src were set at 100% after being normalized to loading control tubulin.

To test our hypothesis regarding the roles of Fyn and Src in D1-NR2B interaction, we used Fyn- and Src-specific small interfering RNAs (siRNAs) to transfect PFC neurons at 11 DIV. First, we determined the efficiency of Fyn and Src siRNA transfection in cultured PFC neurons. Four hours after siRNA application, the neurons were washed out and then maintained in neurobasal medium (Gibco, Carlsbad, CA) at 37°C/5% CO_2 _for 48 h to allow for detectable knockdown. The efficiencies of the transfections were assessed with the neurons that stained with antibodies of either anti-Fyn or Src. We found that both Fyn and Src kinase expressions were significantly decreased in the siRNA-transfected neurons. As shown in Figure 3, Fyn knockdown caused more than 50% loss of Fyn-positive puncta in dendrites (control: 52.3 ± 2.32, n = 30; Fyn knockdown: 28.6 ± 4.15, n = 20; p < 0.01; Figure 3A and 3C). Src siRNA transfection showed similar efficiency (control: 56.2 ± 2.12, n = 30; Src knockdown: 25.3 ± 1.76, n = 30; p < 0.001, Figure 3B and 3D). The siRNA transfections also resulted in significant reductions of fluorescence intensity in dendrites in the immunostaining of both Fyn (control: 74,609 ± 5758, n = 30; Fyn knockdown: 37,738 ± 7658, n = 20; p < 0.01; Figure 3A and 3C) and Src (control: 67,844 ± 8,519, n = 30; Src knockdown: 48,418 ± 7427, n = 30; p < 0.01; Figure 3B and 3D). The efficiencies of Fyn and Src siRNA transfections were further confirmed using Western blotting. Consistently, both kinases were significantly reduced about 50% in protein expression (n = 3; p < 0.01; Figure 3E-H). In contrast, the Src expression in Fyn siRNA transfection and Fyn expression in Src siRNA transfection were unaltered, demonstrating the siRNA knockdown specificity.

**Figure 4:**
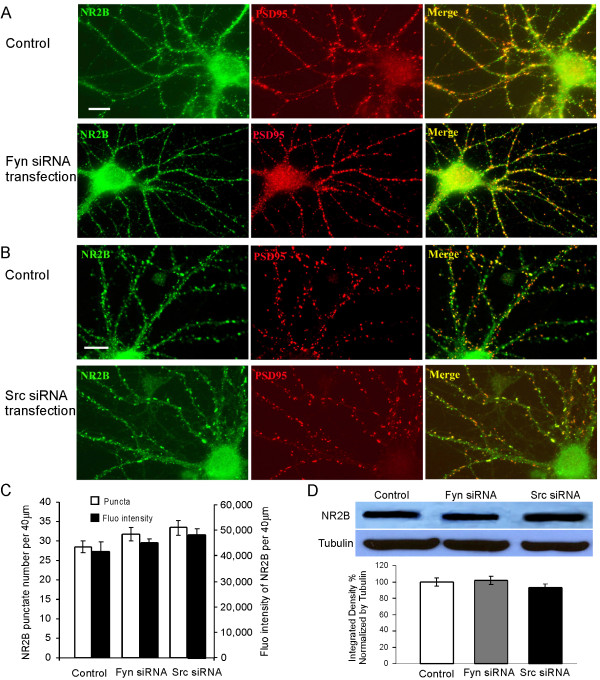
**Src and Fyn knockdowns have no effect on the overall expression or localization and clustering of NR2B subunit at basal conditions**. (A) 11 DIV PFC neurons were transfected with Fyn siRNA for 48 h and stained for endogenous NR2B (green) and PSD95 (red). Scale bars = 10 μm. (B) 11 DIV PFC neurons were transfected with Src siRNA for 48 h and stained for endogenous NR2B (green) and PSD95 (red). Scale bars = 10 μm. (C) Quantification of total NR2B immunofluorescence staining showed that neither Fyn nor Src had a detectable effect on total NR2B puncta number or immunofluorescence intensity compared with the control group (p > 0.05). Data represent mean ± SEM. (D) Total protein levels of NR2B were determined by Western blotting after Fyn and Src siRNA transfection in cultured PFC neurons. The blots were stripped and reprobed with antitubulin as loading control. Control protein levels of NR2B were set at 100% after being normalized to loading control tubulin (p > 0.05).

Recent studies have emphasized the importance of Src family kinases in regulation of NMDA receptors in the CNS, possibly in enhancing NMDA receptor function [25,26]. However, the effects of Src family kinases on NMDA receptor expression in cultured neurons remain unexplored. To assess the functions of Fyn and Src in the regulation of NMDA receptor, PFC neuronal cultures were fixed and stained with antibody against NR2B after Fyn or Src knockdown with siRNA transfection. Interestingly, as shown in Figure 4, we found that neither Fyn nor Src knockdown affected the basal NR2B expression in cultured PFC neurons in either puncta number (28.6 ± 1.5 in control vs 31.7 ± 1.8 in Fyn knockdown, n = 30, p > 0.05; and 33.5 ± 1.98 in Src knockdown, n = 45, p > 0.05) or fluorescence intensity (control: 41,670 ± 8,875, n = 30; Fyn knockdown: 45,005 ± 4133, n = 30, p > 0.05; Src knockdown: 48,515 ± 7,408, n = 45, p > 0.05; Figure 4A-C). However, when both kinases were knockdown, all neurons died after the treatment. The unaltered NR2B expressions in siRNA knockdown were also confirmed by Western blotting. The homogenates from high-density cultured PFC neurons with either Fyn or Src knockdown were prepared and the protein levels of NR2B subunits were found to be unchanged under either Fyn or Src knockdown conditions, with no statistical difference (n = 3; Fyn knockdown vs control, p = 0.137; Src knockdown vs control, p = 0.100; Figure 4D).

### D1 receptor-mediated increases in NR2B expression and surface insertion depend on the Fyn, but not the Src, signaling pathway

**Figure 5:**
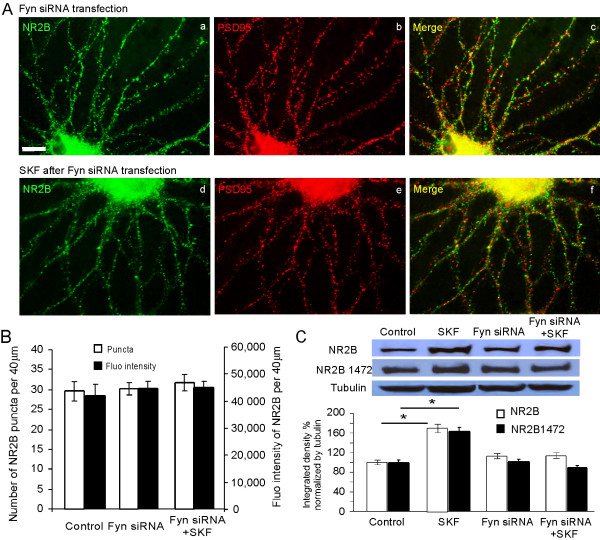
**D1 agonist induces no changes in NR2B expression after Fyn knockdown**. Immunostaining revealed that neither NR2B nor PSD95 expression was changed by treatment with SKF-81297 (10 μM) in Fyn siRNA-transfected PFC neurons (d, e, f) compared with control (a, b, c). Scale bar = 10 μm. (B) Quantification of total NR2B immunofluorescence staining revealed that SKF-81297 (10 μM) had no clear effects on NR2B puncta number or fluorescence intensity in the dendrites of Fyn siRNA-transfected neurons compared with control group (p > 0.05). (C) Total protein levels of NR2B, which were determined by Western blotting after Fyn siRNA transfection followed by SKF treatment in cultured PFC neurons, were consistently unaltered by treatment with SKF-81297 (10 μM, p > 0.05) although SKF-81297 (10 μM) induced a significant increase in NR2B expression in the control condition (*p < 0.05). Similarly, in neurons with Fyn siRNA, NR2B Tyr1472 phosphorylation level was not changed by treatment with SKF-81297 (10 μM, p > 0.05). Integrated intensity analysis was performed using Image J software and the control protein level of NR2B or the Tyr1472 phosphorylation was set to 100% after being normalized by loading control tubulin.

**Figure 6:**
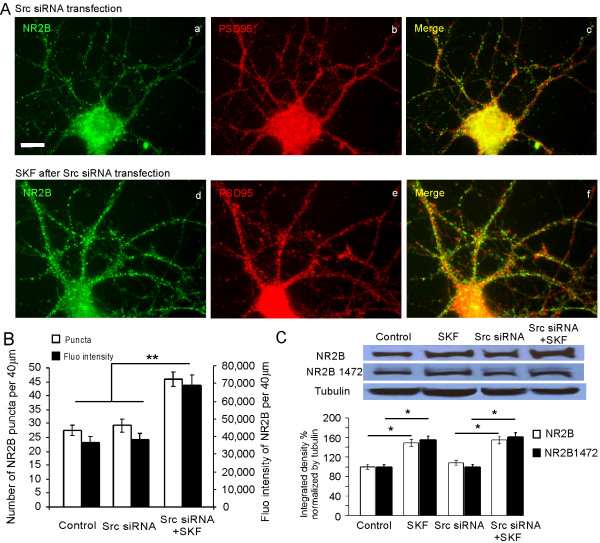
**Src knockdown has no effect on the D1 receptor-mediated localization and clustering of NR2B**. (A) Immunostaining revealed that NR2B expression was significantly increased by SKF-81297 (10 μM) treatment in Src siRNA-treated PFC neurons, compared with the control group. Scale bar = 10 μm. (B) Quantification of total NR2B immunofluorescence staining revealed that SKF treatment increased total NR2B puncta number and fluorescence intensity in dendrites even after Src siRNA transfection, compared with control. Results are presented as the mean number of total NR2B puncta and mean intensity of fluorescence (**p < 0.01). Statistical analysis was performed by t test and the data represent mean ± SEM. (C) Similarly, total protein or NR2B Tyr1472 phosphorylation levels of NR2B determined by Western blotting after Src siRNA transfection remained to be significantly altered by SKF-81297 treatment in cultured PFC neurons (* p < 0.05). Control NR2B protein level or NR2B Tyr1472 phosphorylation level was set to 100% after being normalized by loading control tubulin.

We next examined the specific roles of Src and Fyn in D1 receptor-mediated modulation of NMDA receptor trafficking. In our recent study [35], we found that D1 receptor-mediated increase of NR2B insertion in cultured PFC neurons was selectively blocked by Src family kinase inhibitor PP2 but not by its analogue PP3. Because PP2 is a general inhibitor of Src family kinases, here we further differentiated the specific roles of the Src- or Fyn-dependent signaling pathways in the D1-mediated regulation of NMDA receptor trafficking by using either Fyn or Src siRNA knockdown in the cultured prefrontal neurons. As described above, 72 hrs after the transfection of Fyn or Src siRNA, PFC neuron cultures were treated with vehicle DMSO (0.1%) or the D1 receptor agonist SKF-81297 (10 μM) for 10 min and were then restored to 37°C/5% CO_2 _in normal medium for another 15 min. As shown in Figure 5, the D1 receptor-mediated effects on NR2B expression were completely blocked by Fyn knockdown in both puncta number (control: 29.6 ± 2.5, n = 30; SKF after Fyn knockdown: 30.2 ± 1.6, n = 25, p > 0.05) and fluorescence intensity (control: 41,876 ± 3,992, n = 30; SKF after Fyn knockdown: 44,433 ± 3,992, n = 25, p > 0.05; Figure 5A and 5B), as well as protein levels of NR2B (n = 3, p = 0.275; Figure 5C). In contrast, the D1 receptor-mediated NR2B expression was unchanged after Src knockdown. D1 receptor stimulation still significantly increased both puncta number (control: 27.6 ± 1.9, n = 15; Src knockdown: 45.9 ± 2.6, n = 25, p < 0.01) and fluorescence intensity (control: 36,661 ± 3618, n = 15; Src knockdown: 68,995 ± 5,773, n = 25, p < 0.01; Figure 6A and 6B), as well as protein expression of NR2B (n = 3, p < 0.01; Figure 6C). These results strongly indicate that Fyn, but not Src, is responsible for the D1 receptor-mediated enhancement of NR2B expression in cultured prefrontal neurons.

Previous studies in other brain regions have demonstrated that activation of Src family kinases leads to phosphorylation of NR2B which appears to be important for surface expression of this subunit [23,32]. Therefore, we used a specific antibody to look at the phosphorylation state of the NR2B subunit. We found that knockdown of Fyn by siRNA blocked the phosphorylation of NR2B Tyr1472 after D1 activation (vehicle-transfected and non SKF-81297 treatment control: 100%; vehicle-transfected neurons treated with SKF-81297: 163.4% ± 2.4%, p < 0.05; Fyn siRNA, non SKF-81297 treatment: 101.2% ± 2.5%, p > 0.05; Fyn siRNA, SKF-81297: 92.2% ± 2.6%, p > 0.05; n = 3 in each group) (Figure 5C) while siRNA knockdown of Src had no effect on D1-induced NR2B Tyr1472 phosphorylation (vehicle-transfected and non SKF-81297 treatment control: 100%; vehicle-transfected neurons treated with SKF-81297: 156.7% ± 2.5%, p < 0.05; Src siRNA, non SKF-81297 treatment: 99.5% ± 2.5%, p > 0.05; Src siRNA, SKF-81297: 162.2 ± 3.6%, p < 0.05; n = 3 in each group) (Figure 6C). This indicates that under our experimental conditions, Fyn phosphorylates NR2B while Src does not.

**Figure 7:**
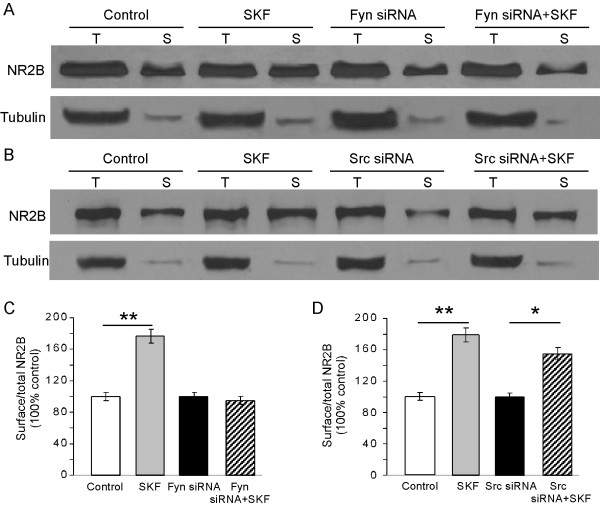
**Fyn, but not Src, affects the surface NR2B expression after D1 receptor stimulation**. (A and B) Surface biotinylation of NMDA receptors in high-density PFC neurons at 14 DIV. PFC neurons at12 DIV were transfected with Fyn siRNA for 48 h. The PFC neurons at DIV 14 were treated with DMSO (0.1%, lanes 1 and 2); SKF-81297 (10 μM, lanes 3 and 4); Fyn (A) or Src (B) knockdown PFC neurons treated with DMSO (0.1%, lanes 5 and 6); or Fyn (A) or Src (B) knockdown neurons treated with SKF-81297 (10 μM, lanes 7 and 8). After surface receptor biotinylation, 20% of the lysis supernatant was used to detect the total (T) proteins. The remaining 80% of the supernatant was incubated with NeurAvidin Agarose beads and purified as surface (S) proteins. After SDS-PAGE the proteins were immunoblotted for NR2B and β-tubulin. (C and D) Quantification of surface NR2B expression. Surface NR2B was corrected by total NR2B to calculate the surface/total ratio and the control group was set to 100% for normalization. Fyn, but not Src, knockdown appeared to be effective in blocking the surface insertion of NR2B mediated by D1 stimulation (* p < 0.05, ** p < 0.01).

Finally, we used a surface biotinylation assay to examine whether Fyn and Src affect NR2B trafficking under this condition. We found that treatment of high-density PFC cultured neurons with D1 receptor agonist SKF-81297 significantly increased the ratio of surface/total NR2B expression compared with the control group (n = 3, p < 0.01; Figure 7). As Figure 4 shows, neither Fyn nor Src knockdown affected total protein levels of NR2B expression (Fyn knockdown: n = 3, p = 0.45; Src knockdown: n = 3, p = 0.10). In contrast to the almost unaltered surface NR2B expression under conditions of activation of D1 agonist SKF-81297 after Src knockdown (n = 3, p < 0.05; Figure 7B and 7D), however, the D1 effects on the surface NR2B expression were completely blocked by Fyn knockdown (n = 3, p = 0.448; Figure 7A and 7C). Taken together, these results demonstrate that D1 receptor activation specifically increases surface NR2B expression by a Fyn-dependent signaling pathway, whereas Src is not involved.

## Discussion

We have demonstrated that D1 receptor activation-mediated enhancement of NR2B expression, in the cultured PFC neurons depends on the Fyn but not Src signaling pathway by taking the advantage of siRNA knockdown. Our data provide evidence of a novel molecular mechanism involved in the D1-NMDA interaction in prefrontal neurons.

Interaction of D1 and NMDA receptors in PFC has long been proposed to contribute to synaptic plasticity and to cognitive functions. Pharmacologic blockade of either D1 or NMDA receptor results in many cognitive deficits such as decreases in spatial working memory [1,36,37]. Physiologically, D1 receptor stimulation potentiates the NMDA receptor responses in PFC neurons in vitro [5,9-11,38]. The D1-dependent NMDA receptor enhancement in PFC appears to be critical both for physiological regulation of synaptic strength in working memory function [6,39] and for disorders such as drug addiction [40] and schizophrenia [3].

NMDA receptors are essential for long-lasting changes in synaptic efficacy such as long-term plasticity. Considerable evidence indicates that NMDA receptors are not static residents in synapses but instead can move in and out [5], whereby they may regulate receptor function and synaptic plasticity [23]. On the other hand, neuronal activity drives not only local receptor synthesis but also receptor insertion into the plasma membrane, lateral diffusion between synaptic and extrasynaptic sites, and receptor endocytosis [23,41,42]. Thus, the notion has been emerging that activity-dependent NMDA receptor trafficking provides a potentially powerful mechanism for the regulation of synaptic efficacy and remodeling. It is increasingly appreciated that NMDA receptor trafficking dysregulation may contribute to neuropsychiatric disorders such as drug addiction [43], Alzheimer's disease [44], and schizophrenia [45]. Therefore, understanding the role of D1 receptor stimulation in modulating NMDA receptor trafficking is vital in order to elucidate the mechanisms underlying synaptic plasticity and neuropsychiatric disorders.

Increasing evidence suggests that Src family kinases play essential roles in the regulation of NMDA receptor by D1 receptor activation [46,47]. Our recent study [35] shows that D1 receptor-mediated increase in NR2B surface expression and synaptic function in PFC was selectively blocked by Src family kinase inhibitor PP2. The Src family of protein tyrosine kinases expressed in the nervous system includes Src, Fyn, Yes, Lck and Lyn. Because of the high homology between the family members, determining the role of specific members of the Src family kinases in the regulation of NMDA receptor trafficking has been challenging. Whereas Src has been clearly shown to play a role in the regulation of NMDA receptors [28,48] and in the induction of long-term potentiation in hippocampal CA1 [28,49], gene-targeted deletion of Src failed to show apparent neurological phenotypes [28,29]. Several confounding factors may explain these conflicting results. Pharmacological tools for specific blockade of Src or Fyn are lacking. In addition, Src family kinases may have both specific and overlapping functions in various physiological processes [50]. Therefore molecular redundancy among Src family kinases may lead to functional compensation, thus confounding the phenotype of knockout mice. In other words, knockout mice might not be the ideal tools to study the specific functions of Src family members. To determine whether and which specific Src kinases have a role in D1-induced NMDA receptor trafficking, we took the siRNA approach. Indeed, as recent study [51] has shown, siRNA knockdown approach has some advantages over the gene knockout approach in studying the function of closely related family members such as MAGUKS in glutamate receptor trafficking. Using the siRNA approach, we found that dopamine D1-activation induced NMDA receptor trafficking depends on Fyn, but not Src signaling pathway.

NMDA receptor tyrosine phosphorylation at position 1472 (Tyr1472) might stabilize NMDA receptors on the cell surface, thereby increasing NMDA receptor responses. Accordingly, Src family kinase activation might inhibit NMDA receptor endocytosis [32,52]. We observed the effect of D1 receptor stimulation on the surface expression of the NR2B subunit in cultured PFC neurons, consistent with previous studies [21,22]. D1 receptor agonist treatment leads to significant increase in NR2B dendritic localization and colocalization with PSD95. Fyn knockdown effectively blocks the NMDA receptor increase after D1 activation, whereas Src knockdown exhibits no clear effect, suggesting that Fyn, but not Src, is involved in D1 receptor modulation of NMDA receptor expression. Our data agree with numerous recent studies in which Tyr1472 phosphorylation was found to be required for proper NR2B localization at synapses in the striatum [21,46], hippocampus [32], amygdala [53], and PFC [22]. It should be mentioned that although we focused on the NR2B subunit in this study, we found that D1 also increased NR1 subunits (Li et al., unpublished observations). This is important as there are functional differences among NMDA receptors as recent work has demonstrated [54].

Our RNAi efficiency is similar to that of others as reported in neurons [55]. The reason why that the protein level of Src or Fyn is reduced to ~50% of the control is a matter of speculation. Due to the variance in protein stability and cell systems, the efficacy of siRNA also varies. As for why the remaining Fyn is insufficient for enhancing NMDA receptor trafficking after D1 activation, we speculate that Fyn might have some important functions in cell survival and cell physiology [56], and for the economy of cells, it is not unreasonable to think that cell survival has a higher priority than the receptor trafficking.

### Conclusions

Our findings demonstrate that dopamine receptors may regulate synaptic plasticity by modulating NMDA receptor synaptic expression through a Fyn-dependent signaling pathway. Considering that NMDA receptor activity alterations are involved in the clinical features of schizophrenia and drug abuse, our study not only provides insight into the roles of Src family kinases in NMDA receptor trafficking but also offers the possibility of generating new pharmacologic reagents targeting Fyn kinase signaling pathway. This intervention could be promising for psychiatric disorders involving D1-NMDA interaction, such as schizophrenia and drug addiction.

## Methods

### Chemicals and antibodies

The chemicals used for treatment of PFC neurons were purchased from the following sources: selective D1 agonist SKF-81297, D1 antagonist SCH-23390, and dimethyl sulfoxide (DMSO) were purchased from Sigma-Aldrich (St. Louis, MO). Antibodies used for immunofluorescence and Western blotting included mouse anti-NR2B (1:2000, Millipore, Billerica, MA), rabbit phospho-Y1472 NR2B (1:500, PhosphoSolutions, Aurora, CO), rabbit anti-PSD95 (1:1000, Frontier Science Co., Hokkaido, Japan), rat anti-D1 (1:1000, Sigma), rabbit anti-Fyn (1:1000, Millipore), mouse anti-Src (1:1000, Millipore), and mouse antitubulin (1:10000, Millipore). The primary antibody dilutions listed above were for Western blotting. For immunofluorescence staining, dilutions were all 1:800. The following reagents were obtained from Jackson ImmunoResearch (West Grove, PA): Cy3-conjugated donkey antimouse secondary antibody (1:800), FITC-conjugated donkey anti-mouse secondary antibody (1: 800), Cy3-conjugated donkey anti-rabbit secondary antibody (1:800), FITC-conjugated donkey anti-rabbit secondary antibody (1:800), peroxidase-conjugated goat anti-mouse secondary antibody (1:1000), and peroxidase-conjugated goat anti-rabbit secondary antibody (1:1000).

### PFC cultures

Timed pregnant rats and adult rats (3 months old) were purchased from Marshall Farms (New York, NY) and the animal procedures were in strict accordance with the National Institute of Health (NIH) animal use guideline. The experimental protocols were approved by the Institutional Animal Care and Use Committee at Drexel University College of Medicine. Primary neuron cultures were prepared from embryonic day 20 rat PFC. Briefly, PFC tissues were isolated and dissociated with trypsin (Gibco, Carlsbad, CA) at 37°C, filtered by cell filter (BD Falcon, San Jose, CA), rinsed with Neurobasal medium (Gibco, Carlsbad, CA) supplemented with B27and L-glutamine(Gibco, Carlsbad, CA) and 5% fetal bovine serum (FBS). Cultures were plated at 2 × 10^5 ^cells per well on poly-l-lysine coated glass coverslips in 6-well plates (BD Falcon, San Jose, CA) and kept in Neurobasal medium supplemented with B27, L-glutamine, and 5% FBS at 37°C/5% CO_2 _for 4 h. Cells were then maintained in Neurobasal medium supplemented with B27, and L-glutamine without FBS at 37°C/5% CO_2_. Cells were fed 2 times per week, with one third of the media changed each time. Cultured PFC neurons were used between 14 and 18 days in vitro (DIV) for the studies described.

### siRNA transfection

Src and Fyn siRNAs were purchased from Millipore, including pKD-Fyn-v6 (mammalian Fyn siRNA expression plasmid) and pKD-Src-v2 (mammalian Src siRNA expression plasmid).

Src siRNA sequence:

Sense strand:

5'-TGGTGGCCTACTACTCCAAACTTCAAGAGAGTTTGGAGTAGTAGGCCACCATTTTTG-3'

Antisense strand:

5'-AATTCAAAAATGGTGGCCTACTACTCCAAACTCTCTTGAAGTTTGGAGTAGTAGGCCACCA-3'

Fyn siRNA sequence:

Sense strand:

5'-ACATCGTCACCGAGTATGTGATTCAAGAGATCATATACTCGGTGACGATGTTTTTTG-3'

Antisense strand:

5'-AATTCAAAAAACATCGTCACCGAGTATATGATCTCTTGAATCACATACTCGGTGACGATGT-3'

The siRNAs were used to transfect PFC neurons (750 ng per well) with GeneSilencer siRNA transfection reagent from Genlantis (San Diego, CA) when cultures reached 70% confluence. Neurons in the control conditions were transfected with the same volume of transfection reagent without siRNA. After 4 h, neurons were washed with serum-free Neurobasal medium, and neurons were maintained in Neurobasal medium supplemented with B27 and L-glutamine at 37°C/5% CO_2 _for 48 to 72 h to allow for detectable knockdown prior to the treatment with other pharmacologic reagents. The efficiencies of siRNA transfection were assessed by both immunostaining and Western blotting with antibodies against Fyn or Src, whereas the antibody specificities were demonstrated by re-probing the Src and Fyn, respectively.

### Pharmacologic treatments of PFC neurons

To assess the roles of Fyn and Src in D1 receptor-mediated modulation of NMDA receptor, PFC neurons with or without transfection were treated with the selective D1 receptor agonist SKF-81297 (10 μM) for 10 min or pretreated with selective D1 receptor antagonist SCH-23390 (15 μM) followed by SKF-81297 (10 μM) for 10 min. The cultured neurons were then restored to normal medium at 37°C/5% CO_2 _for another 15 min to allow the trafficking of NMDA receptors. For control, the same volume of DMSO (0.1%) was added as vehicle and the cultured neurons were subjected to the same conditions. Precautions were taken to protect samples from light exposure and oxidation. The treated PFC neurons were used for both immunofluorescent staining and Western blotting.

### Immunofluorescent staining of PFC neurons

PFC neurons were fixed by methanol at -20 °C for 5 min and rinsed with 0.2% Triton X-100 (Dow Chemical, Midland, MI) in phosphate-buffered saline (PBS) to permeabilize the plasma membrane. Coverslips were blocked with 10% bovine serum albumin in PBS for 1 h at room temperature. Double immunofluorescent stainings were conducted with antibodies against NMDA receptors (NR2B), D1 receptor, Fyn, Src, or postsynaptic marker PSD95, followed by appropriate secondary antibodies. Images were acquired using a Zeiss Axiovert 200 M inverted microscope with Axiovision software (Zeiss Microscopy, Jena, Germany). All analysis and quantifications were performed using the NIH Image J software. The dendritic segments in neurons (40 μm) were randomly selected for puncta analysis. The average intensity of fluorescence staining and the punctate number of NR2B/Fyn/Src were quantified using the NIH Image J software and were blindly confirmed by other researchers in the laboratory. Results were presented as the mean number of total puncta or mean intensity of fluorescence ± standard error of the mean (SEM). Statistical analysis was performed using one-way analysis of variance (ANOVA) followed by Tukey multiple-comparison tests for several experimental groups. Images were prepared for printing with Adobe Photoshop (San Jose, CA) and Canvas (ACD Systems, Ltd., Victoria, BC, Canada).

### Biotinylation assay

The biotinylation was performed as described with minor modification [57]. After transfection with Fyn or Src siRNA, neurons were treated with the pharmacologic agents as described above. For cell surface receptor biotinylation, neurons were rinsed twice with ice-cold 0.1 M PBS for 1 min each and were then incubated with 1.5 mg/mL sulfo-NHS-SS-Biotin (Pierce, Rockford, IL, USA) in 0.1 M PBS for 20 min at 4 °C. The sulfo-NHS biotin was quenched with PBS containing 50 mM glycine. After washing twice with ice-cold 0.1 M PBS (5 min each), neurons were then lysed in radioimmunoprecipitation assay (RIPA) buffer. The homogenates were centrifuged at 14,000 g for 10 min at 4 °C. The resulting supernatant volume was measured and 20% of it separated as the total (T) protein. The remaining 80% of the supernatant was incubated with NeutrAvidin agarose beads (Pierce) overnight at 4 °C. NeutrAvidin agarose beads were washed with RIPA buffer and then spun down for 2 min at 5000 rpm, which was repeated 3 times. Finally, the biotinylated proteins were eluted from the NeutrAvidin by incubation with 2 × sample SDS-PAGE buffer at 95°C for 5 min and used as surface (S) proteins. Western blotting was performed using antibody against NR2B. Data were quantified by comparing the ratio of surface biotinylated to total input protein and normalized to control group as percentages.

### Crude Synaptosome (P2) Preparation

Crude synaptosome (P2) was prepared from rat frontal cortex as previously described [58,59] with minor modification. Cerebral cortices were collected and homogenized in 40 ml buffered sucrose (0.32 M Sucrose/1 mM NaHCO3). The homogenate was centrifuged at 1,400 g for 10 min, then the pellet (P1) was discarded, while the supernatant (S1) was saved and centrifuged at 13,800 g for 10 min. The resulting pellet (P2) was crude synaptosome, which was resuspended by RIPA buffer and resolved by electrophoresis. The crude synaptosome was used in Figure 1B, whereas homogenates were used in all other experiments for the data exhibited in Figures 2, 3, 4, 5, 6 and 7.

### Western blotting

Proteins were isolated from adult rat PFC or high-density prefrontal neuronal cultures with RIPA buffer. After centrifugation at 14,000 rpm at 4 °C for 15 min, the supernatants were resolved by electrophoresis on 7.5% polyacrylamide gels and transferred onto nitrocellulose membranes. The membranes were blocked in 5% nonfat milk in TBS for 1 h and were incubated with the following antibodies overnight at 4 °C: mouse anti-NR2B, rabbi anti-PSD95, rat anti-D1, rabbit anti-Fyn, mouse anti-Src, and mouse antitubulin. After incubation with appropriate horseradish peroxidase-conjugated secondary antibodies, antigens were identified by enhanced chemiluminescence reagents [60]. Blots were scanned and quantified using Image J software. Control protein levels were set at 100% after being normalized to loading control tubulin. All other values were normalized as percentages of control. Each set of experiments was repeated at least 3 times to reduce interblot variability. Images were prepared for printing with Adobe Photoshop and Canvas.

## Competing interests

The authors declare that they have no competing interests.

## Authors' contributions

JLH carried out the biochemical experiments, data analysis, and wrote the manuscript. GL did cell cultures, carried out immunostaining, participated in biochemical experiments and data analysis. YCL participated in immunostaining and data analysis. WJG helped the manuscript preparation and prepared the manuscript for publication. YQH conceived and designed the study and revised the manuscript. All authors read and approved the final manuscript.

## Supplementary Material

Additional file 1 **D1-mediated Increase of NMDA Receptors May Require Protein Synthesis and Depends on PKA Signaling**. The file includes graphs which show the effects of protein synthesis inhibitor anisomycin and PKA inhibitor KT5720 and PKC inhibitor Go6983 on D1-mediated increase of NR2B cluster number and fluorescence. Click here for file
