# Supplementary material for: Dopamine D1 receptor-mediated NMDA receptor insertion depends on Fyn but not Src kinase pathway in prefrontal cortical neurons
Source: Mol Brain. 2010 Jun 22;3:20. doi: 10.1186/1756-6606-3-20 (PMC2902469; doi:10.1186/1756-6606-3-20)
Supplement: Additional file 1 — D1-mediated Increase of NMDA Receptors May Require Protein Synthesis and Depends on PKA Signaling. The file includes graphs which show the effects of protein synthesis inhibitor anisomycin and PKA inhibitor KT5720 and PKC inhibitor Go6983 on D1-mediated increase of NR2B cluster number and fluorescence. [file 1756-6606-3-20-S1.DOC]

Hu et al Additional Files

**Dopamine D1 receptor-mediated NMDA receptor insertion depends on Fyn but not Src kinase pathway in prefrontal cortical neurons**

Jian-Li Hu ^1*^, Gang Liu ^1^, Yan-Chun Li ^1^, Wen-Jun Gao, Yue-Qiao Huang ^*^

*Department of Neurobiology and Anatomy, Drexel University College of Medicine, 2900 W. Queen Lane, Philadelphia, PA 19129, USA*

**D1-mediated Increase of NMDA Receptors May Require Protein Synthesis and Depends on PKA Signaling**

Because the total expression of NR2B was significantly increased by D1 stimulation, we therefore tested the effect of anisomycin, a protein synthesis inhibitor, as well PKA and PKC inhibitors, on the D1-induced increase in total NR2B subunits. Whereas the cluster numbers were significantly increased in SKF-81297 (1 μM; n = 20, p < 0 .01; Additional Figure 1), pretreatment with anisomycin (10 μM) blocked the change in cluster number (n = 20, p>0.05). Interestingly, inclusion of PKA inhibitor KT5720 (5 μM), but not PKC inhibitor Go6983 (20 μM), also effectively blocked the D1 effect on total NR2B expression (cluster numbers: SKF + PKA inhibitor, n = 20, p > 0.05; SKF + PKC inhibitor, n = 20, p < 0.05).


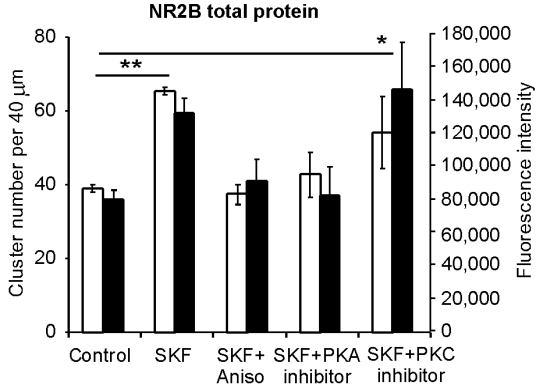


**Additional Figure 1.** Protein synthesis inhibitor anisomycin (10 μM) and PKA inhibitor KT5720 (5 μM) but not PKC inhibitor Go6983 (20 μM) completely blocked D1 effect on NR2B cluster numbers and fluorescence intensity (n *=* 20, * p<0.05; ** p < 0.01).
